# Supplementary material for: Pyruvate Dehydrogenase Kinase 1 inhibition mediated oxidative phosphorylation enhancement in cartilage promotes osteoarthritis progression
Source: BMC Musculoskelet Disord. 2023 Jul 20;24:597. doi: 10.1186/s12891-023-06585-6 (PMC10357736; doi:10.1186/s12891-023-06585-6)
Supplement: Supplementary file 1 — Additional file 1. [file 12891_2023_6585_MOESM1_ESM.zip › MF.Gsea.1653627316242/GOMF_SINGLE_STRANDED_RNA_BINDING.html]

Details for gene set GOMF\_SINGLE\_STRANDED\_RNA\_BINDING[GSEA]

|  || Dataset | 55457\_collapsed\_to\_symbols.cls.cls #OA\_versus\_normal.cls.cls #OA\_versus\_normal\_repos |
| Phenotype | cls.cls#OA\_versus\_normal\_repos |
| Upregulated in class | normal |
| GeneSet | GOMF\_SINGLE\_STRANDED\_RNA\_BINDING |
| Enrichment Score (ES) | -0.37929508 |
| Normalized Enrichment Score (NES) | -1.5343697 |
| Nominal p-value | 0.008237232 |
| FDR q-value | 0.16353172 |
| FWER p-Value | 1.0 |
Table: GSEA Results Summary

  

Fig 1: Enrichment plot: GOMF\_SINGLE\_STRANDED\_RNA\_BINDING      
 Profile of the Running ES Score & Positions of GeneSet Members on the Rank Ordered List

  

| SYMBOL | TITLE | RANK IN GENE LIST | RANK METRIC SCORE | RUNNING ES | CORE ENRICHMENT || 1 | TLR7 | toll like receptor 7 [Source:HGNC Symbol;Acc:HGNC:15631] | 61 | 0.971 | 0.0323 | No |
| 2 | POLR2G | RNA polymerase II subunit G [Source:HGNC Symbol;Acc:HGNC:9194] | 183 | 0.830 | 0.0545 | No |
| 3 | PPIE | peptidylprolyl isomerase E [Source:HGNC Symbol;Acc:HGNC:9258] | 334 | 0.733 | 0.0707 | No |
| 4 | DDX60 | DExD/H-box helicase 60 [Source:HGNC Symbol;Acc:HGNC:25942] | 1385 | 0.445 | 0.0058 | No |
| 5 | CBX4 | chromobox 4 [Source:HGNC Symbol;Acc:HGNC:1554] | 1397 | 0.443 | 0.0219 | No |
| 6 | ADARB2 | adenosine deaminase RNA specific B2 (inactive) [Source:HGNC Symbol;Acc:HGNC:227] | 1828 | 0.381 | 0.0029 | No |
| 7 | CBX6 | chromobox 6 [Source:HGNC Symbol;Acc:HGNC:1556] | 1875 | 0.376 | 0.0136 | No |
| 8 | IFIH1 | interferon induced with helicase C domain 1 [Source:HGNC Symbol;Acc:HGNC:18873] | 2204 | 0.338 | 0.0009 | No |
| 9 | LACTB2 | lactamase beta 2 [Source:HGNC Symbol;Acc:HGNC:18512] | 2604 | 0.290 | -0.0191 | No |
| 10 | RBM7 | RNA binding motif protein 7 [Source:HGNC Symbol;Acc:HGNC:9904] | 2620 | 0.288 | -0.0093 | No |
| 11 | ATXN1 | ataxin 1 [Source:HGNC Symbol;Acc:HGNC:10548] | 2862 | 0.264 | -0.0180 | No |
| 12 | FXR1 | FMR1 autosomal homolog 1 [Source:HGNC Symbol;Acc:HGNC:4023] | 3012 | 0.249 | -0.0202 | No |
| 13 | DDX1 | DEAD-box helicase 1 [Source:HGNC Symbol;Acc:HGNC:2734] | 3305 | 0.221 | -0.0345 | No |
| 14 | ZFR2 | zinc finger RNA binding protein 2 [Source:HGNC Symbol;Acc:HGNC:29189] | 3309 | 0.220 | -0.0264 | No |
| 15 | AGO1 | argonaute RISC component 1 [Source:HGNC Symbol;Acc:HGNC:3262] | 3430 | 0.208 | -0.0278 | No |
| 16 | TLR8 | toll like receptor 8 [Source:HGNC Symbol;Acc:HGNC:15632] | 3579 | 0.196 | -0.0319 | No |
| 17 | SNRPC | small nuclear ribonucleoprotein polypeptide C [Source:HGNC Symbol;Acc:HGNC:11157] | 3649 | 0.190 | -0.0300 | No |
| 18 | KHDRBS1 | "KH RNA binding domain containing, signal transduction associated 1 [Source:HGNC Symbol;Acc:HGNC:18116]" | 3748 | 0.182 | -0.0307 | No |
| 19 | MSI1 | musashi RNA binding protein 1 [Source:HGNC Symbol;Acc:HGNC:7330] | 3893 | 0.165 | -0.0356 | No |
| 20 | L1TD1 | LINE1 type transposase domain containing 1 [Source:HGNC Symbol;Acc:HGNC:25595] | 4056 | 0.151 | -0.0425 | No |
| 21 | DHX58 | DExH-box helicase 58 [Source:HGNC Symbol;Acc:HGNC:29517] | 4130 | 0.143 | -0.0427 | No |
| 22 | MCRS1 | microspherule protein 1 [Source:HGNC Symbol;Acc:HGNC:6960] | 4286 | 0.129 | -0.0499 | No |
| 23 | AGO4 | argonaute RISC component 4 [Source:HGNC Symbol;Acc:HGNC:18424] | 4346 | 0.124 | -0.0498 | No |
| 24 | A1CF | APOBEC1 complementation factor [Source:HGNC Symbol;Acc:HGNC:24086] | 4368 | 0.123 | -0.0467 | No |
| 25 | KHDRBS2 | "KH RNA binding domain containing, signal transduction associated 2 [Source:HGNC Symbol;Acc:HGNC:18114]" | 4550 | 0.109 | -0.0566 | No |
| 26 | PTBP1 | polypyrimidine tract binding protein 1 [Source:HGNC Symbol;Acc:HGNC:9583] | 4650 | 0.100 | -0.0605 | No |
| 27 | ZC3H14 | zinc finger CCCH-type containing 14 [Source:HGNC Symbol;Acc:HGNC:20509] | 4896 | 0.080 | -0.0766 | No |
| 28 | EIF4H | eukaryotic translation initiation factor 4H [Source:HGNC Symbol;Acc:HGNC:12741] | 5118 | 0.062 | -0.0914 | No |
| 29 | PIWIL1 | piwi like RNA-mediated gene silencing 1 [Source:HGNC Symbol;Acc:HGNC:9007] | 6144 | -0.016 | -0.1707 | No |
| 30 | DDX58 | DExD/H-box helicase 58 [Source:HGNC Symbol;Acc:HGNC:19102] | 6480 | -0.041 | -0.1953 | No |
| 31 | RBMS1 | RNA binding motif single stranded interacting protein 1 [Source:HGNC Symbol;Acc:HGNC:9907] | 7121 | -0.090 | -0.2417 | No |
| 32 | HNRNPC | heterogeneous nuclear ribonucleoprotein C [Source:HGNC Symbol;Acc:HGNC:5035] | 7437 | -0.114 | -0.2619 | No |
| 33 | LONP1 | "lon peptidase 1, mitochondrial [Source:HGNC Symbol;Acc:HGNC:9479]" | 7531 | -0.121 | -0.2646 | No |
| 34 | EIF4A3 | eukaryotic translation initiation factor 4A3 [Source:HGNC Symbol;Acc:HGNC:18683] | 7646 | -0.130 | -0.2685 | No |
| 35 | AQR | aquarius intron-binding spliceosomal factor [Source:HGNC Symbol;Acc:HGNC:29513] | 7654 | -0.131 | -0.2640 | No |
| 36 | FMR1 | FMRP translational regulator 1 [Source:HGNC Symbol;Acc:HGNC:3775] | 7944 | -0.151 | -0.2808 | No |
| 37 | EIF4B | eukaryotic translation initiation factor 4B [Source:HGNC Symbol;Acc:HGNC:3285] | 8051 | -0.159 | -0.2830 | No |
| 38 | RBMS2 | RNA binding motif single stranded interacting protein 2 [Source:HGNC Symbol;Acc:HGNC:9909] | 8373 | -0.184 | -0.3010 | No |
| 39 | EXOSC10 | exosome component 10 [Source:HGNC Symbol;Acc:HGNC:9138] | 8404 | -0.186 | -0.2963 | No |
| 40 | RBPMS | "RNA binding protein, mRNA processing factor [Source:HGNC Symbol;Acc:HGNC:19097]" | 8547 | -0.197 | -0.2999 | No |
| 41 | PABPC4 | poly(A) binding protein cytoplasmic 4 [Source:HGNC Symbol;Acc:HGNC:8557] | 8741 | -0.212 | -0.3068 | No |
| 42 | RBMS3 | RNA binding motif single stranded interacting protein 3 [Source:HGNC Symbol;Acc:HGNC:13427] | 8981 | -0.230 | -0.3167 | No |
| 43 | ZFR | zinc finger RNA binding protein [Source:HGNC Symbol;Acc:HGNC:17277] | 9104 | -0.240 | -0.3170 | No |
| 44 | DLX2 | distal-less homeobox 2 [Source:HGNC Symbol;Acc:HGNC:2915] | 9168 | -0.245 | -0.3126 | No |
| 45 | LSM14A | LSM14A mRNA processing body assembly factor [Source:HGNC Symbol;Acc:HGNC:24489] | 9428 | -0.267 | -0.3226 | No |
| 46 | HNRNPU | heterogeneous nuclear ribonucleoprotein U [Source:HGNC Symbol;Acc:HGNC:5048] | 9847 | -0.306 | -0.3435 | No |
| 47 | IGHMBP2 | immunoglobulin mu DNA binding protein 2 [Source:HGNC Symbol;Acc:HGNC:5542] | 10248 | -0.346 | -0.3615 | No |
| 48 | ELAVL4 | ELAV like RNA binding protein 4 [Source:HGNC Symbol;Acc:HGNC:3315] | 10412 | -0.362 | -0.3605 | No |
| 49 | DHX9 | DExH-box helicase 9 [Source:HGNC Symbol;Acc:HGNC:2750] | 10627 | -0.386 | -0.3624 | Yes |
| 50 | CNBP | CCHC-type zinc finger nucleic acid binding protein [Source:HGNC Symbol;Acc:HGNC:13164] | 10707 | -0.394 | -0.3536 | Yes |
| 51 | TIA1 | TIA1 cytotoxic granule associated RNA binding protein [Source:HGNC Symbol;Acc:HGNC:11802] | 10855 | -0.411 | -0.3494 | Yes |
| 52 | IFIT5 | interferon induced protein with tetratricopeptide repeats 5 [Source:HGNC Symbol;Acc:HGNC:13328] | 11240 | -0.462 | -0.3617 | Yes |
| 53 | AGO2 | argonaute RISC catalytic component 2 [Source:HGNC Symbol;Acc:HGNC:3263] | 11246 | -0.463 | -0.3444 | Yes |
| 54 | HNRNPF | heterogeneous nuclear ribonucleoprotein F [Source:HGNC Symbol;Acc:HGNC:5039] | 11339 | -0.476 | -0.3334 | Yes |
| 55 | LARP4 | La ribonucleoprotein 4 [Source:HGNC Symbol;Acc:HGNC:24320] | 11700 | -0.533 | -0.3412 | Yes |
| 56 | SSB | small RNA binding exonuclease protection factor La [Source:HGNC Symbol;Acc:HGNC:11316] | 11955 | -0.584 | -0.3387 | Yes |
| 57 | U2AF2 | U2 small nuclear RNA auxiliary factor 2 [Source:HGNC Symbol;Acc:HGNC:23156] | 12226 | -0.652 | -0.3349 | Yes |
| 58 | PABPC3 | poly(A) binding protein cytoplasmic 3 [Source:HGNC Symbol;Acc:HGNC:8556] | 12257 | -0.663 | -0.3120 | Yes |
| 59 | PUS1 | pseudouridine synthase 1 [Source:HGNC Symbol;Acc:HGNC:15508] | 12260 | -0.664 | -0.2868 | Yes |
| 60 | ILF3 | interleukin enhancer binding factor 3 [Source:HGNC Symbol;Acc:HGNC:6038] | 12353 | -0.692 | -0.2676 | Yes |
| 61 | HNRNPA1 | heterogeneous nuclear ribonucleoprotein A1 [Source:HGNC Symbol;Acc:HGNC:5031] | 12356 | -0.693 | -0.2413 | Yes |
| 62 | HNRNPDL | heterogeneous nuclear ribonucleoprotein D like [Source:HGNC Symbol;Acc:HGNC:5037] | 12522 | -0.759 | -0.2253 | Yes |
| 63 | HNRNPH1 | heterogeneous nuclear ribonucleoprotein H1 [Source:HGNC Symbol;Acc:HGNC:5041] | 12554 | -0.776 | -0.1981 | Yes |
| 64 | PABPC1 | poly(A) binding protein cytoplasmic 1 [Source:HGNC Symbol;Acc:HGNC:8554] | 12562 | -0.778 | -0.1690 | Yes |
| 65 | CBX8 | chromobox 8 [Source:HGNC Symbol;Acc:HGNC:15962] | 12586 | -0.787 | -0.1408 | Yes |
| 66 | DDX11 | DEAD/H-box helicase 11 [Source:HGNC Symbol;Acc:HGNC:2736] | 12607 | -0.803 | -0.1117 | Yes |
| 67 | JMJD6 | "jumonji domain containing 6, arginine demethylase and lysine hydroxylase [Source:HGNC Symbol;Acc:HGNC:19355]" | 12658 | -0.832 | -0.0839 | Yes |
| 68 | DAZAP1 | DAZ associated protein 1 [Source:HGNC Symbol;Acc:HGNC:2683] | 12686 | -0.854 | -0.0535 | Yes |
| 69 | AGO3 | argonaute RISC catalytic component 3 [Source:HGNC Symbol;Acc:HGNC:18421] | 12726 | -0.888 | -0.0227 | Yes |
| 70 | DDX3X | DEAD-box helicase 3 X-linked [Source:HGNC Symbol;Acc:HGNC:2745] | 12788 | -0.949 | 0.0087 | Yes |
Table: GSEA details [plain text format]

  

Fig 2: GOMF\_SINGLE\_STRANDED\_RNA\_BINDING      
 Blue-Pink O' Gram in the Space of the Analyzed GeneSet

  

Fig 3: GOMF\_SINGLE\_STRANDED\_RNA\_BINDING: Random ES distribution      
 Gene set null distribution of ES for **GOMF\_SINGLE\_STRANDED\_RNA\_BINDING**
